# Optometric challenges in Sierra Leone

**DOI:** 10.7189/jogh.14.03038

**Published:** 2024-09-20

**Authors:** Bryce St. Clair, Mary Araba Otoo, Anny Shi

**Affiliations:** 1 Department of Ophthalmology, The Johns Hopkins Wilmer Eye Institute, Baltimore, Maryland, USA; 2 Cherish Eyesight & Vision Inc., Chicago, Illinois, USA; 3 The Johns Hopkins University, Baltimore, Maryland, USA

In Sierra Leone, the limited workforce of eye care providers underscores the problem of preventable blindness that affects the nation. With fewer than 15 eye care professionals serving a population of approximately eight million people, the scarcity of skilled practitioners significantly hampers accessibility and quality of eye care across the country [1]. This shortage is especially critical in Sierra Leone, one of the world's least-developed nations, facing health care challenges such as geographical barriers, high out-of-pocket expenditures, a shortage of skilled medical personnel, and subpar quality of medical services [1]. Compounding these difficulties, health resources are unevenly distributed with a concentration of referral hospitals and over half of the workforce centred in the urban area of Freetown, the nation's capital [1]. The consequences are severe, with a staggering 91.5% of all blindness in Sierra Leone estimated to be avoidable and 58.2% considered treatable. Attempts have been made to address the absence of local eye care providers such as creating Ophthalmic Community Health Officers for screening purposes [2]. Although this may appear to be a viable solution, the bottleneck of services remains with the limited number of independent, professionally trained eye doctors within the country. This stark reality is underscored by the mere five optometrists and six ophthalmologists tasked with addressing the entire country's eye health needs [1,2]. In light of such insufficiency, a substantial number of individuals suffer from entirely preventable vision impairments, perpetuating a cycle of unnecessary blindness.

Like many other West African nations, Sierra Leone lacks the necessary infrastructure to produce optometric providers [3]. The nearest institutions that train optometrists are in Ghana, over 1700 km away, graduating 105 professionals annually. Despite Ghana’s better standing, trained professionals remain insufficient for the growing population with ocular diseases. Increasing training opportunities is imperative for diversifying the health workforce and alleviating personnel shortages in Sierra Leone.

Unlike ophthalmologists, who primarily focus on performing ocular surgeries to manage conditions like cataracts, glaucoma, and ocular misalignment, optometrists work independently, treating glaucoma, ocular infections, and providing primary eye care as well as providing refractive error correction through glasses and contact lenses. Because optometrists do not perform surgeries, they do not require a specialised surgical team or other supporting staff to carry out their responsibilities. This independence allows optometrists to practice without the need for additional trained professionals. Additionally, optometry does not necessitate specialised surgical training, resulting in a shorter duration of education. This shorter educational path eliminates the added years of training that might discourage potential providers from pursuing this field.

Sierra Leone endured a 10-year civil war, which exacerbated the country’s pervasive poverty as well as impacted various aspects of its infrastructure, including the health care system [4]. In low-income countries, poverty has been linked to higher rates of visual impairment due to limited access to essential eye care services [5]. In order to enhance accessibility, innovative financing solutions like microfinance may be implemented, which has demonstrated advantages at fostering economic resilience and increasing household incomes in Sierra Leone [6]. The nation grapples with ongoing challenges in diagnosing and treating preventable diseases, issues that are less debilitating in more developed nations [7].

The absence of infrastructure to train primary care providers, including those specialising in eye care, exacerbates conditions that could otherwise be managed through preventative medicine. This situation leads to downstream effects, particularly in eye care, resulting in blindness among individuals. People with vision loss are at higher risk of accidents and injuries due to decreased visual acuity. Literature reveals that individuals with visual impairments are 1.9 times more likely to suffer multiple falls compared to those with normal vision [8]. Vision impairment can contribute to accidental injury as well as job loss, placing an undue burden on the community.

The scarcity of health care providers in Sierra Leone results from various interconnected issues. Limited access to education due to financial constraints and inadequate infrastructure impedes many from pursuing medical training [9,10]. Additionally, the phenomenon of ‘brain drain’, where skilled professionals seek better prospects abroad, significantly depletes the health care workforce.

The country faces an uphill battle due to inadequate health care infrastructure, low retention rates of trained professionals, and limited career prospects. Addressing this scarcity requires multifaceted approaches, including improving educational accessibility, investing in health care infrastructure, and offering better incentives and working conditions to retain health care workers. Mitigating brain drain and creating opportunities for professional growth within Sierra Leone are also crucial to augmenting the health care workforce and bolstering the nation's health care system. Potential strategies include promoting robust education and research opportunities to incentivise skilled personnel to stay and requiring government efforts to allocate resources to educational institutions. Additionally, developing career opportunities aligned with professionals’ skills, offering competitive salaries and benefits, and supporting entrepreneurship can collectively foster a conducive environment for professionals to remain in Sierra Leone, contributing to national development.

The scarcity of eye care providers in Sierra Leone has a profound impact on marginalised populations, particularly those residing in remote areas and facing economic hardship. These communities often confront significant challenges in accessing essential eye care services due to the shortage of eye care professionals. As a result, individuals within these marginalised groups may be affected by undiagnosed or untreated eye conditions, potentially leading to vision impairment or even blindness [1,2].

This shortage further exacerbates the vulnerability of marginalised populations to eye-related health issues. Factors such as inadequate nutrition, poor health literacy, and exposure to environmental risks make these communities even more susceptible to eye problems [11,12]. The malnutrition rates of Sierra Leone are among the highest in the world, leading to a variety of ocular problems due to nutritional deficiencies [13]. Additionally, the majority of rural households in Sierra Leonne lack access to clean water, worsening the prevalence of eye infections and diseases [14]. Moving forward, implementing comprehensive public health initiatives aimed at improving socioeconomic conditions, enhancing access to clean water and nutritious food, and expanding affordable eyecare services can serve as interventions that address these root causes of visual impairment.

The lack of routine, preventative health eye exams, treatments, or access to corrective lenses due to the scarcity of eye care providers contributes to worsening eye health conditions among these marginalised groups [15]. One key to addressing low levels of ocular health literacy in Sierra Leone involves staging community outreach and education programmes aimed at explaining ocular pathologies and their treatments. Effectively mitigating preventable blindness through interventions would require integration into local communities and fostering awareness through engagement initiatives [16].

While specific data on the consequences of vision impairment in Sierra Leone is scarce, parallels can be drawn from the experiences in other countries. The World Health Organization reports global productivity loss due to unaddressed vision loss amounts to 410.7 billion US dollars annually, far exceeding the cost of addressing this unmet need. It can be inferred that impaired vision or blindness greatly impacts the daily lives and future economic success of marginalised individuals in underdeveloped countries like Sierra Leone. The limitations resulting from untreated eye conditions hinder their educational opportunities, employment prospects, and social integration, perpetuating cycles of poverty and exclusion within these communities. For instance, studies have shown that children with poor academic performance had worse visual health than those with good academic standing [17]. The disproportionate burden of vision-related health challenges faced by marginalised populations underscores the urgent need to address the shortage of eye care providers to ensure equitable access to essential eye care services for all members within Sierra Leone’s society.

Despite the challenges posed by the shortage of eye care providers in Sierra Leone, the opportunity to create significant positive changes within the nation is immense. Integrating innovative technologies such as artificial intelligence (AI) into primary care settings could revolutionise eye care delivery in Sierra Leone. AI-enabled screening for common ocular pathologies like glaucoma, diabetic retinopathy, and cataracts, supported by reliable internet access and trained personnel, has the potential to expand diagnostic capabilities across Sierra Leone. Similarly, leveraging telehealth and mobile health applications could expand access to remote areas, though challenges in continuity of care and regulatory hurdles remain. In Sierra Leone, as in many low-resource settings, there is a fragmentation and uneven distribution of health care services, which lead to gaps in accessibility and efficiency [1]. Thus, integrating such optometric services into existing health care infrastructure and establishing effective referral pathways between primary care clinics and eye care centres can foster a more cohesive and accessible health care approach.

While short-term solutions like deploying mobile training units and forming partnerships with non-profit organisations or foreign institutions can provide immediate relief, the sustainability of such interventions depends on fostering local expertise and infrastructure [18]. One such successful initiative is the Mozambique Eyecare project, which established the Mozambique School of Optometry, graduating more than 300 optometry providers and addressing the shortage of eye health professionals in Mozambique as well as neighbouring African countries [19]. Therefore, educating prospective optometrists domestically holds great promise for the nation, offering significant advantages particularly for marginalised communities already facing considerable challenges. By training health care professionals within the country, underserved populations can expect enhanced accessibility to health care services, resulting in notable improvements in their overall well-being and health care outcome.
